# Chronic Exposure to Fine Particles and Mortality: An Extended Follow-up of the Harvard Six Cities Study from 1974 to 2009

**DOI:** 10.1289/ehp.1104660

**Published:** 2012-03-28

**Authors:** Johanna Lepeule, Francine Laden, Douglas Dockery, Joel Schwartz

**Affiliations:** 1 Department of Environmental Health, and; 2 Department of Epidemiology, Harvard School of Public Health, Boston, Massachusetts, USA; 3 Channing Laboratory, Brigham and Women’s Hospital, Harvard Medical School, Boston, Massachusetts, USA

**Keywords:** air pollution, cohort studies, concentration–response, follow-up studies, lag, lung cancer, mortality, particles, PM_2.5_, threshold

## Abstract

Background: Epidemiologic studies have reported associations between fine particles (aerodynamic diameter ≤ 2.5 µm; PM_2.5_) and mortality. However, concerns have been raised regarding the sensitivity of the results to model specifications, lower exposures, and averaging time.

Objective: We addressed these issues using 11 additional years of follow-up of the Harvard Six Cities study, incorporating recent lower exposures.

Methods: We replicated the previously applied Cox regression, and examined different time lags, the shape of the concentration–response relationship using penalized splines, and changes in the slope of the relation over time. We then conducted Poisson survival analysis with time-varying effects for smoking, sex, and education.

Results: Since 2001, average PM_2.5_ levels, for all six cities, were < 18 µg/m^3^. Each increase in PM_2.5_ (10 µg/m^3^) was associated with an adjusted increased risk of all-cause mortality (PM_2.5_ average on previous year) of 14% [95% confidence interval (CI): 7, 22], and with 26% (95% CI: 14, 40) and 37% (95% CI: 7, 75) increases in cardiovascular and lung-cancer mortality (PM_2.5_ average of three previous years), respectively. The concentration–response relationship was linear down to PM_2.5_ concentrations of 8 µg/m^3^. Mortality rate ratios for PM_2.5_ fluctuated over time, but without clear trends despite a substantial drop in the sulfate fraction. Poisson models produced similar results.

Conclusions: These results suggest that further public policy efforts that reduce fine particulate matter air pollution are likely to have continuing public health benefits.

All-cause, cardiopulmonary, cardiovascular, and lung-cancer mortality have been associated with chronic air pollution exposure in prospective studies that controlled for individual covariates (Abbey et al. 1999; Beelen et al. 2008b; Beeson et al. 1998; Cao et al. 2011; Dockery et al. 1993; Eftim et al. 2008; Filleul et al. 2005; Gehring et al. 2006; Katanoda et al. 2011; Laden et al. 2006; Miller et al. 2007; Nafstad et al. 2004; Ostro et al. 2010; Pope et al. 2002; Puett et al. 2009; Yorifuji et al. 2011). The studies that specifically considered lung-cancer mortality associations with fine particles (aerodynamic diameter < 2.5 µm; PM_2.5_), all found positive associations (Beelen et al. 2008b; Dockery et al. 1993; Laden et al. 2006; McDonnell et al. 2000), although this association was only statistically significant (*p* < 0.05) in the American Cancer Society study (ACS) (Pope et al. 2002; Turner et al. 2011).

Although compelling evidence supports the harmful effects of PM_2.5_ on longevity, concerns have been raised regarding the sensitivity of the results to model specifications. In particular, Moolgavkar (2005, 2007) suggested that covariates may not be proportional and hence were not controlled for properly in proportional hazards models; that the concentration–response relation may not be linear; and that there are few observations at levels as low as or below the current World Health Organization and U.S. Environmental Protection Agency (EPA) air quality standards. In addition, the relative toxicity of particle elements is still controversial, and most of the recent reduction in PM_2.5_ concentrations in the United States has come from sulfate control. Hence it is of interest whether the concentration–response curve has changed over time as particle composition has changed. Health impact assessments in the United States assume that health benefits of reducing particles are only fully realized after 20 years (U.S. EPA 2010), so examination of the lag between exposure and mortality is also relevant for consideration of changes in the standard.

Our goal was to test the robustness of the association between chronic exposure to PM_2.5_ and mortality observed in the original study (Dockery et al. 1993), and the first extended follow-up of the Harvard Six Cities study (Laden et al. 2006) by replicating the analyses using 11 additional years of follow-up with exposures well below the U.S. annual standard (15 µg/m^3^) (U.S. EPA 1997). We examined different lags of exposure, tested the shape of the PM_2.5_ concentration–mortality relationship, tested for changes in this slope over time, and relaxed the proportion assumption by allowing the effects of covariates to vary each year. We reexamined the association of PM_2.5_ with specific causes of death such as lung cancer and examined the effects of PM_2.5_ depending on participants’ chronic conditions and smoking status.

## Methods

*Study population.* The Harvard Six Cities study population has been previously described (Dockery et al. 1993). Briefly, adults were randomly sampled from six cities in the eastern and midwestern United States between 1974 and 1977: in 1974, Watertown, Massachusetts; in 1975, Kingston and Harriman, Tennessee, and specific census tracts of St. Louis, Missouri; in 1976, Steubenville, Ohio, and Portage, Wyocena, and Pardeeville, Wisconsin; and in 1977, Topeka, Kansas. Information on age, sex, weight, height, educational level, smoking history, hypertension, and diabetes was collected by questionnaire at enrollment. All participants underwent spirometry tests at enrollment (Dockery et al. 1985) and chronic obstructive pulmonary disease (COPD) was defined as having

(FEV_1_ ÷ FVC) < 70%,

where FEV_1_ is forced expiratory volume in 1 sec, and FVC is forced vital capacity. This analysis, as in the previous analyses, was restricted to 8,096 white participants with acceptable pulmonary function measurements. The study was approved by the Harvard School of Public Health Human Subjects Committee and all participants signed an informed consent before participation.

*Mortality follow-up.* Vital status and cause of death were determined by searching the National Death Index (NDI) for calendar years 1979–2009. Deaths before the NDI started in 1979 were identified by next of kin and Social Security records, and the cause of death was determined by a certified nosologist who reviewed death certificates (Dockery et al. 1993).

*Survival time.* Survival times were calculated from enrollment until death or the end of follow-up (31 December 2009). For the 6 participants who were lost to follow-up before 1979, the censored survival times were calculated from enrollment to date of the last follow-up contact plus 6 months or the first day of the NDI (1 January 1979), whichever came first. For each cause of death category, participants who died from another cause were censored at time of death.

*Air pollution estimates.* Annual PM_2.5_ concentration was assigned for each participant until death or censoring. PM_2.5_ concentration was measured in the participant’s city by a centrally located monitor from 1979 to 1986–1988, depending on the city (Dockery et al. 1993). Therefore, the study has no spatial contrast on the within-city scale. PM_2.5_ concentrations for the years before monitoring started were assumed to be equal to the earliest monitored year. From the end of monitoring until 1998, PM_2.5_ concentration was estimated from PM_10_ (aerodynamic diameter < 10 µm) data from U.S. EPA monitors and visibility (extinction) data from the National Weather Service (Laden et al. 2006). From 1999 through 2009, direct measurements of PM_2.5_ were available from U.S. EPA monitors. For sensitivity analyses, we also predicted PM_2.5_ for 1999–2009 (correlation between predicted and measured was 0.97) using the formula applied to derive exposure estimates during the earlier period when PM_2.5_ was not measured.

*Statistical analysis.* We first replicated the original analysis separately for all-cause mortality, cardiovascular mortality as coded by the *International Classification of Diseases, 9th Revision* [ICD-9; World Health Organization (WHO) 1977] or the *10th Revision* (ICD-10; WHO 1992), 400.0–440.9, I10.0–I70.9, respectively, lung-cancer mortality (ICD-9 162, ICD-10 C33.0–C34.9), and COPD mortality (ICD-9 490.0–496.0, ICD-10 J40.0–J47.0) for the 36-year follow-up from 1974 to 2009 using a Cox proportional hazards model with follow-up time as the time scale (Dockery et al. 1993; Laden et al. 2006). PM_2.5_ was included in each model as an annual time-dependent variable. The model was stratified by sex, age (1-year intervals) and time in the study (1-year intervals), so that each age/sex group had its own baseline hazard for each year of follow-up. The analysis was adjusted for potential confounders collected at baseline: smoking status (never, former, current), cumulative smoking (pack-years included separately for current and former smokers), educational level (< high-school, ≥ high school), and a linear and quadratic term for body mass index (BMI; kilograms per meter squared), using the Cox proportional hazards model formulated as follows:

*h_i s_*(*t*) = *h*_0_ *_s_*(*t*) exp[β_1_*X_i_* + β_2_*Z_i_*(*t*)], [1]

where *h_i_* is the instantaneous hazard probability of death for subject *i* in stratum *s* (defined by sex, age, and time in the study), *h*_0_*_s_*(*t*) is the baseline hazard function, *X_i_* is the vector of time-independent variables, and *Z_i_*(*t*) is the vector of time-dependent variables. We evaluated models with 1-year (i.e., exposure during the year before death or censure) to 5-year lagged moving averages and chose the best fit model using Akaike’s information criterion (AIC) (Akaike 1973). The best fit moving average was determined from participants who survived at least 5 years from enrollment, so that AIC criteria were evaluated among populations with comparable sizes. We then estimated mortality rate ratios (RR) associated with PM_2.5_ exposure during the best fit moving average on the whole sample size. Once the best exposure window was determined, we fit a penalized spline model using a cubic regression spline with 12 knots to estimate the shape of the concentration–response relation, and chose the optimal degree of freedom by minimizing AIC and evaluated nonlinearity with a Wald test. We investigated whether PM_2.5_ advanced date of death for participants with chronic conditions at enrollment. We also investigated the potential for effect modification of PM_2.5_ on mortality by smoking status at enrollment using interaction terms between such variables and PM_2.5_. Finally, we tested the hypothesis that the effect of PM_2.5_ changed over time by dividing the follow-up into four equally spaced time periods and testing interactions between period and PM_2.5_.

*Sensitivity analyses.* We performed sensitivity analyses using a second-degree polynomial distributed lag model to allow the effects of PM_2.5_ exposure to be distributed from 1 to 5 years before death or censor (Lepeule et al. 2006; Schwartz 2000); using predicted PM_2.5_ concentrations after 1999 instead of the measured PM_2.5_; considering only deaths from natural causes, with external causes of deaths (ICD-9 E800–E999, ICD-10 S00–T88 and V00–Y99) being censored at time of death; and considering only deaths that occurred in the state where the participants lived at enrollment. We next investigated the robustness of the results to alternative modeling assumptions by using a Poisson model with dummy variables for each year of follow-up, which is equivalent to a piecewise exponential proportionate hazard model with the baseline hazard changing each year (Laird and Oliver 1981):

log µ*_it_* = log *E_it_* + γ*_t_T_t_* + β_1_*X_i_* + β_2_*Z_i_*(*t*), [2]

where µ*_it_* is the expected value of the death indicator for subject *i* at time *t*, *E_it_* is the exposure duration of subject *i* at time *t* (log *E_it_* being the offset), *T_t_* is the vector of dummy variables for time by 1 year (piece-wise baseline hazard), *X_i_* is the vector of the time-independent covariates, and *Z_i_*(*t*) is the vector of time-dependent variables. Using this Poisson survival analysis, we first compared the results to the Cox model and then relaxed the proportionate hazard assumption for sex, education, and cumulative smoking by including interaction terms of these variables with each year of follow-up. As an alternative to the previous analyses (Dockery et al. 1993; Laden et al. 2006), we used age in 5-year groups as the time scale, and adjusted the model for time trends (linear term). For specific causes of death, convergence issues led us to group age by 10 years. We then fit penalized spline models. Because RRs may vary over time and period-specific RRs may be biased, we used the Poisson model to calculate adjusted survival curves (Hernan 2010). We included product terms between PM_2.5_ and time in model 2 [Equation 2], thereby allowing the effect of PM_2.5_ to flexibly vary from year to year. We then predicted the survival probability for each year of follow-up for each participant under three scenarios using concentrations of PM_2.5_ throughout the entire follow-up period equal to 10, 15, or 20 µg/m^3^.

*p*-Values < 0.05 were considered statistically significant. All analyses were repeated separately for all- and specific-causes of deaths. Analyses were conducted with SAS software, version 9.2 (SAS Institute Inc., Cary, NC) and R statistical software, version 2.12.2 (R Foundation for Statistical Computing, Vienna, Austria).

## Results

*Study population.* The 8,096 participants were 25–74 years of age at enrollment (mean ± SD, 49.6 ± 13.4) and 54.7% were female. More than half of the participants had a high school degree or higher, 35.8% were current smokers, and 23.9% were former smokers and the average BMI was 25.8 ± 4.5. As for chronic conditions, 17.8% reported hypertension, 11.6% COPD, and 6.9% diabetes.

**Figure 1:**
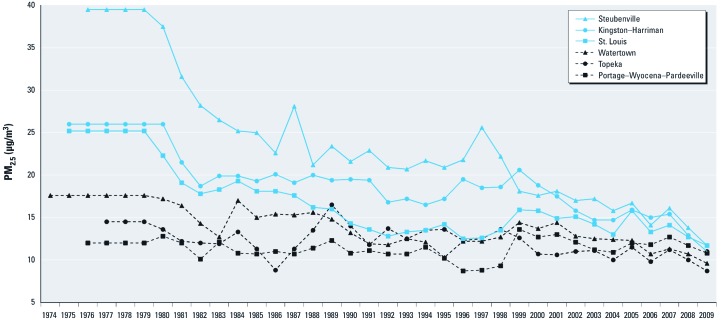
Annual mean PM_2.5_ levels during 1974–2009 in the Harvard Six Cities study.

*Mortality rates and PM_2.5_ levels.* At the end of 2009, there were 212,067 person-years of follow-up and 55.5% of the participants had died, among whom 40.8% died from cardiovascular diseases, 7.8% from lung cancer, and 5.5% from COPD (Table 1). Overall, PM_2.5_ concentration decreased during the study period (Figure 1). After 1998, annual average levels declined by 1.8 µg/m^3^ in St. Louis and by 10.5 µg/m^3^ in Steubenville, whereas levels increased by 1.5 µg/m^3^ in the Portage–Wyocena–Pardeeville area. Since 2000, all the cities experienced average PM_2.5_ levels < 15 µg/m^3^ except Kingston–Harriman and Steubenville, which had average concentrations of ≤ 18 µg/m^3^.

**Table 1:** Number of participants, mortality, and average PM_2.5_ levels in the Harvard Six Cities study, 1974–2009.

Characteristic	Six cities (combined)	Steubenville	Kingston–Harriman	St. Louis	Watertown	Topeka	Portage–Wyocena–Pardeeville
Participants (n)	8,096	1,346	1,258	1,292	1,332	1,238	1,630
Person-years (n)	212,067	33,276	33,067	32,225	36,818	32,877	43,804
Cause of death							
All causes [n (%)]	4,495 (55.5)	822 (61.1)	733 (58.3)	827 (64.0)	700 (52.6)	617 (49.8)	796 (48.8)
Cardiovascular (%)	40.8	45.3	41.1	42.2	39.3	37.4	38.6
Lung cancer (%)	7.8	9.0	8.0	8.7	6.6	7.3	6.8
COPD (%)	5.5	4.9	7.0	5.1	4.9	7.3	4.6
1974–2009 average of individual PM2.5 concentrations	15.9	23.6	19.1	16.7	14.0	12.2	11.4

*Association between PM_2.5_ and mortality.* Using the Cox proportional hazards model, statistically significant associations between PM_2.5_ exposure and all-cause, cardiovascular, and lung-cancer mortality were observed (Table 2). The AIC indicated lag 1 (i.e., exposure during the previous year) to be the best fit exposure window for all-cause mortality [see Supplemental Material, Table 1 (http://dx.doi.org/10.1289/ehp.1104660)]. For cause-specific mortality, the best fit moving average differed between the Cox and the Poisson regressions. Because the differences in AIC were very small between the 1- and 5-year moving averages for both the Cox and Poisson regressions, we chose the longer of the two moving averages to produce more stable results, specifically, a 1- to 3-year moving average for cardiovascular and lung-cancer mortality, and a 1- to 5-year moving average for COPD mortality. Each 10-µg/m^3^ increase in PM_2.5_ was associated with a 14% increased risk of all-cause death [95% confidence interval (CI): 7%, 22%], a 26% increase in cardiovascular death (95% CI: 14%, 40%), and a 37% increase in lung-cancer death (95% CI: 7%, 75%). For both all-cause mortality and specific causes of death, the model fit was better without the spline (*p*-values between 0.24 and 0.43), indicating a linear relationship with PM_2.5_. Results restricted to participants with chronic conditions at enrollment (i.e., hypertension, COPD, or diabetes) were consistent with those estimated for all participants (Table 2). Although, the interaction between smoking status and PM_2.5_ was not statistically significant, there was a trend for a stronger estimated effect of PM_2.5_ on mortality in current and former smokers. However, positive associations between PM_2.5_ and all-cause and cardiovascular mortality were still evident in never smokers. RR for PM_2.5_ fluctuated over time for all-cause mortality and specific causes of death, without clear trends (Table 2).

**Table 2:** Adjusted^a^ association between PM_2.5_^b^ and mortality, for the 8,096 participants and certain subpopulations of the Harvard Six Cities study, 1974–2009.

Cause of death/stratum-specific estimates according to characteristics at enrollment		n participants (n person-years)	RR (95% CI) for 10-µg/m^3^ increase in PM_2.5_
All-cause			8,096 (212,067)		1.14 (1.07, 1.22)
	Chronic conditionsc				
	Hypertension		1,439 (30,540)		1.17 (1.03, 1.32)
	COPD		942 (17,723)		1.09 (0.95, 1.26)
	Diabetes		563 (11,473)		1.04 (0.85, 1.27)
	Smoking status (p-interaction = 0.58)				
	Never smoker		3,265 (90,372)		1.09 (0.98, 1.21)
	Former smoker		1,934 (48,049)		1.17 (1.04, 1.30)
	Current smoker		2,897 (73,646)		1.17 (1.06, 1.28)
	Follow-up period (p-interaction = 0.06)				
	1974–1982		8,096 (58,798)		1.06 (0.96, 1.17)
	1983–1991		7,478 (63,129)		1.32 (1.16, 1.50)
	1992–2000		6,391 (51,800)		1.11 (0.98, 1.27)
	2001–2009		4,910 (38,340)		1.19 (0.91, 1.55)
Cardiovascular			7,961 (195,941)		1.26 (1.14, 1.40)
	Smoking status (p-interaction = 0.45)				
	Never smoker		3,232 (83,861)		1.21 (1.04, 1.41)
	Former smoker		1,891 (44,205)		1.21 (1.02, 1.44)
	Current smoker		2,838 (67,875)		1.36 (1.17, 1.58)
	Follow-up period (p-interaction = 0.07)				
	1974–1982		7,961 (42,672)		1.08 (0.92, 1.27)
	1983–1991		7,478 (63,129)		1.46 (1.21, 1.76)
	1992–2000		6,391 (51,800)		1.30 (1.06, 1.59)
	2001–2009		4,910 (38,340)		1.57 (1.01, 2.43)
Lung cancer			7,961 (195,941)		1.37 (1.07, 1.75)
	Smoking status (p-interaction = 0.15)				
	Never smoker		3,232 (83,861)		1.25 (0.54, 2.89)
	Former smoker		1,891 (44,205)		1.96 (1.29, 2.99)
	Current smoker		2,838 (67,875)		1.25 (0.95, 1.64)
	Follow-up period (p-interaction = 0.19)				
	1974–1982		7,961 (42,672)		1.45 (0.98, 2.15)
	1983–1991		7,478 (63,129)		0.94 (0.58, 1.52)
	1992–2000		6,391 (51,800)		1.54 (0.98, 2.41)
	2001–2009		4,910 (38,340)		2.84 (1.06, 7.59)
COPD			7,805 (180,106)		1.17 (0.85, 1.62)
	Smoking status (p-interaction = 0.35)				
	Never smoker		3,191 (77,422)		0.85 (0.36, 2.02)
	Former smoker		1,847 (40,453)		1.64 (0.92, 2.93)
	Current smoker		2,767 (62,231)		1.10 (0.74, 1.62)
	Follow-up period (p-interaction = 0.35)				
	1974–1982		7,805 (26,837)		0.79 (0.36, 1.72)
	1983–1991		7,478 (63,129)		1.52 (0.90, 2.56)
	1992–2000		6,391 (51,800)		1.31 (0.74, 2.31)
	2001–2009		4,910 (38,340)		0.68 (0.25, 1.83)
aCox proportional hazards model stratified by sex, age, and time in the study and adjusted for BMI, education, and smoking history. bPM2.5 moving average was 1 year before death or censure for all-cause deaths, 1–3 years for cardiovascular and lung-cancer deaths, and 1–5 years for COPD deaths. cEstimates restricted to participants with the specified chronic condition.					

*Sensitivity analysis.* For both all causes and specific causes of death, the cumulative effects estimated from the polynomial distributed lag model were similar to the effect estimates obtained with the selected moving averages (Table 2). However, the five lags were too correlated (between 0.90 and 0.96) to disentangle the relative importance of each one. Using predicted PM_2.5_ instead of measured PM_2.5_ for exposures after 1999, excluding the 138 deaths from external causes and excluding the 702 participants who died in a state other than the state where they lived at enrollment, did not change the results (data not shown) except for the lung-cancer mortality association with PM_2.5_, which was slightly attenuated (increased risk of 28%; 95% CI: –2%, 67% compared with 37%; 95% CI: 7%, 75%) when the 702 participants were excluded.

**Figure 2:**
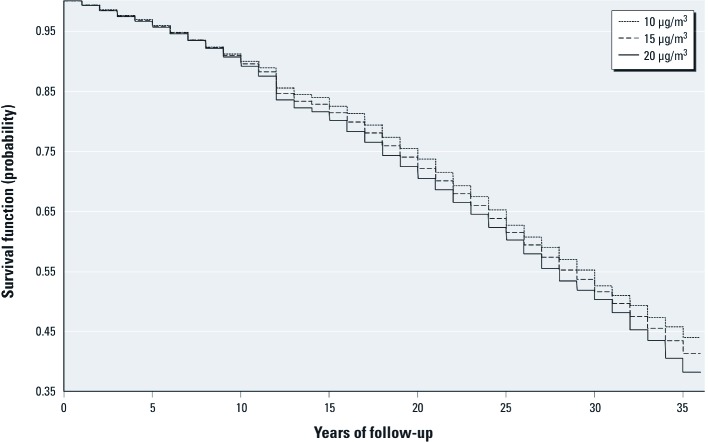
Survival probabilities under three hypothetical scenarios: participants of the Harvard Six Cities study are exposed to 10, 15, or 20 µg/m^3^ PM_2.5_ during the entire follow-up period of 1974–2009.

With the Poisson framework, using basic assumptions, relaxed proportionate hazard assumption for covariates, or age as the time scale, the effect estimates and *p*-values fluctuated slightly but without any notable change in the results compared with estimates from the Cox models for all-cause mortality and for specific-causes of death (data not shown). The penalized spline models also indicated linear concentration–response relationships without a threshold for PM_2.5_ and mortality from all-causes and specific-causes [see Supplemental Material, Figure 1 (http://dx.doi.org/10.1289/ehp.1104660)]. With the Poisson survival analysis, we predicted survival assuming every participant was exposed to a constant concentration of PM_2.5_ (10, 15, or 20 µg/m^3^) during the entire follow-up period. Adjusted for individual covariates, the lowest PM_2.5_ concentration was associated with the highest survival (Figure 2). The three adjusted survival curves showed that the proportionate hazard was a reasonable assumption for PM_2.5_ and that PM_2.5_ effects were quite stable over time.

## Discussion

Including more recent observations with PM_2.5_ exposures down to 8 µg/m^3^, we continued to find a statistically significant association between chronic exposure to PM_2.5_ and all-cause and cardiovascular mortality. Furthermore, in the present extended follow-up, PM_2.5_ exposure was also statistically significantly associated with lung-cancer mortality. Our study indicated no sensitivity of the results for all-cause mortality and specific causes of death when we allowed the effects of smoking, education, and sex to vary over time, or when we used age as the time scale instead of follow-up time. Using very flexible modeling assumptions, our results did not show any rationale for change of PM_2.5_ effect size over the whole study period, as indicated by the adjusted survival curves and the lack of a clear interaction of PM_2.5_ with the four study periods. The concentration–response relationship was linear without any threshold, even at exposure levels below the U.S. annual 15-µg/m^3^ standard (U.S. EPA 1997). Taken together with the results of a previous reanalysis of the Harvard Six Cities study (Krewski et al. 2005b), there is evidence for a robust association between chronic PM_2.5_ exposure and early mortality.

*Consistency of the results.* Our results indicated a statistically significant 14% increase in all-cause mortality for a 10-µg/m^3^ annual increase in PM_2.5_, which is similar to the results of the previous follow-ups (Dockery et al. 1993; Laden et al. 2006). The Netherlands Cohort Study on Diet (NLCS–Air) in Europe (Beelen et al. 2008b), the Adventist Study (McDonnell et al. 2000), and the male Health Professionals Follow-up Study in the United States (Puett et al. 2011) did not show statistically significant associations between PM_2.5_ and all-cause mortality. However, our current results are consistent with those from the ACS cohort (Pope et al. 2002), the Nurses’ Health Study (Puett et al. 2009), and the Medicare cohort (Eftim et al. 2008), which indicated mortality increases ranging from 3–26% per 10-µg/m^3^ increase in PM_2.5_.

The 26% increase in cardiovascular mortality for each 10-µg/m^3^ increase in PM_2.5_ exposure during the previous 3 years estimated in this extended follow-up is similar to the previous estimate (Laden et al. 2006). Although the NLCS–Air study (Beelen et al. 2008b) found no statistically significant association, the magnitude of the estimated effect reported here is between the 12% increase estimated for the ACS cohort (Pope et al. 2004) and the 76% increase estimated for the Women’s Health Initiative study (Miller et al. 2007). Puett et al. (2009) also estimated a 100% increase in fatal coronary heart diseases for a 10-µg/m^3^ increase in PM_2.5_ during the prior year. Underlying mechanisms for the effects of PM_2.5_ on cardiovascular mortality are still poorly understood, but changes in vasoconstriction might explain the associations (Anderson et al. 2011).

The previous extended follow-up of the Harvard Six Cities study showed an elevated, but not statistically significant, risk of lung-cancer mortality (Laden et al. 2006), whereas the present extended follow-up estimated a statistically significant 37% increase in lung-cancer mortality (for each 10-µg/m^3^ increase in PM_2.5_), which is greater than that estimated for both the ACS cohort (14%) (Pope et al. 2002) and a Japanese cohort (27%) (Katanoda et al. 2011). Lungs are one of the organs that are most directly affected by particulate air pollution. Fine particles, which may carry toxic chemicals of carcinogenic potential (Laden et al. 2000), can reach lung alveoli where the clearance is slow (Pinkerton et al. 1995) and induce durable pulmonary and systemic inflammation (Riva et al. 2011). Recent findings in the ACS cohort indicated that a 10-µg/m^3^ increase in PM_2.5_ concentration was associated with a statistically significant 15% to 27% increase in lung-cancer mortality in never smokers (Turner et al. 2011). We did not find such an association in our study, which might be due to a lack of statistical power (350 lung-cancer deaths, 26 among never smokers). However, estimated effects of PM_2.5_ on all-cause and cardiovascular mortality were also statistically significant (or borderline significant) in never smokers, and higher in current smokers compared to never or former smokers (Table 2).

Regarding COPD mortality, we found a positive but not statistically significant risk of COPD death associated with PM_2.5_ exposure. In the ACS cohort, Pope et al. (2004) estimated an unexpected inverse association between PM_2.5_ exposure and COPD mortality, whereas Katanoda et al. (2011) estimated an inverse but not statistically significant association between PM_2.5_ and COPD in a Japanese cohort.

*Chronic conditions at enrollment and mortality.* The central deposition of particles in lungs has been shown to be enhanced in COPD patients (Bennett et al. 1997). Although PM_2.5_ has been associated with early mortality in COPD patients (Zanobetti et al. 2008), and ozone has been associated with early mortality in susceptible subjects (i.e., with COPD, diabetes, heart failure, or myocardial infarction) (Zanobetti and Schwartz 2011), our results did not indicate stronger associations in participants with such chronic conditions at enrollment compared with the population as a whole,. This might have been due to a lack of statistical power as few participants had COPD (*n* = 942) or diabetes (*n* = 563) at enrollment.

*Exposure assessment.* Use of outdoor measurements from central monitoring stations as a proxy measure of mean personal exposure to PM_2.5_ is prone to measurement error because the measures do not capture fine spatial contrasts that may occur within a city, which may bias the results. Recent reanalyses of the ACS cohort using land use regression models showed that the impact on the PM_2.5_–mortality association was heterogeneous depending on the city (Krewski et al. 2009). However, other recent studies have suggested that considering a more precise exposure model focused on the home address might not improve health effects estimates in terms of bias and variance (Kim et al. 2009; Lepeule et al. 2010; Szpiro et al. 2011). In the Harvard Six Cities study, there were not enough monitors in the cities to implement a land use regression model.

*Strengths and limitations.* Our results were adjusted for baseline factors, but there is potential for residual confounding for risk factors after enrollment and for unmeasured factors such as occupational exposures or medication use if those factors co-vary with PM_2.5_. Some other limitations are that we did not measure PM_2.5_ in the same locations throughout the study period, that death certificates might have listed misclassified specific causes of death, and that hypertension and diabetes were assessed by questionnaire only. An extensive body of methodological work has been performed regarding the sensitivity of estimated associations between long-term exposure to air pollution and mortality, especially for the ACS and Harvard Six Cities study cohorts. More specifically, it has been shown that results were robust to alternative model specifications, alternative metrics of PM_2.5_, and adjustment for individual and ecological risk factors such as occupational exposures and socioeconomic variables (Krewski et al. 2005a, 2005b). It was also shown that using a spatial covariance structure did not change the results (Pope et al. 2002), but with only six locations, that methodology is not applicable in our study. Whereas the primary analysis from the Harvard Six Cities study (Dockery et al. 1993) estimated associations were based on between–city contrasts in exposure, in the current study, with age used as time scale, the exposure relied on both between– and within–city contrasts, limiting the potential for residual cross-sectional confounding. The strengths of the present study are the randomly sampled participants and its extended follow-up through 2009, which included more observations of participants with lower exposures during recent years and provided more statistical power.

*Critical periods of PM_2.5_ exposure.* Our results indicated that the best fit moving average for PM_2.5_ was 1 year for all-cause mortality. For cardiovascular and lung-cancer mortality, no clear pattern was identified because of the high correlation between PM_2.5_ concentrations in the 5 lagged years tested. These results suggest that PM_2.5_ exposure can act to promote cardiovascular diseases and lung-cancer growth, although the design of this study precludes us from determining whether PM_2.5_ initiates these diseases as suggested by other studies (Beelen et al. 2008a; Beeson et al. 1998). These results agree with the literature (Gehring et al. 2006; Krewski et al. 2009; Puett et al. 2009; Schwartz et al. 2008) and suggest that health improvements can be expected almost immediately after a reduction in air pollution. This conclusion should be taken into account for cost–benefit analyses related to air pollution standards.

*Role of sulfates and public health implications.* Although RRs for PM_2.5_ fluctuated over time, our extended follow-up did not indicate any clear pattern over time during the study period. Between 1979–1988 (Laden et al. 2000) and 2009 (Nehls and Akland 1973), the sulfates/PM_2.5_ ratio for exposures measured for the Harvard Six Cities study dropped between 13% and 54%, depending on the city. If sulfates are unrelated to mortality, as some have argued (Grahame and Schlesinger 2005), the elimination of a substantial fraction of nontoxic material from PM_2.5_ mass should result in a substantial increase in the PM_2.5_ coefficient, which would otherwise have been suppressed by the large fraction of mass that was nontoxic. This was not the case, and hence our results indicate that sulfate particles are about as toxic as the average fine particle. This is consistent with the results of Pope et al. (2007), who found that the 2.5-µg/m^3^ decrease in sulfate particle concentrations observed during an 8-month smelters strike were associated with a 2.5% decrease in the number of deaths in the region. In comparison, a 2.5-µg/m^3^ decrease in PM_2.5_ in our follow-up of the Harvard Six Cities study was associated with a 3.5% reduction in all-cause deaths, but that was for reductions in PM_2.5_ lasting at least a year, not 8 months. Given that there were 2,423,712 deaths in the United States in 2007 (Xu et al. 2010) and that the average PM_2.5_ level was 11.9 µg/m^3^ (U.S. EPA 2011), our estimated association between PM_2.5_ and all-cause mortality implies that a decrease of 1 µg/m^3^ in population-average PM_2.5_ would result in approximately 34,000 fewer deaths per year.

## Conclusion

Including recent observations with PM_2.5_ exposures well below the U.S. annual standard of 15 µg/m^3^ and down to 8 µg/m^3^, the relationship between chronic exposure to PM_2.5_ and all-cause, cardiovascular, and lung-cancer mortality was found to be linear without a threshold. Our results were not sensitive to various model specifications. Furthermore, estimated effects of PM_2.5_ did not change over time, suggesting a stable toxicity of PM_2.5_, even at lower exposure levels and with a lower sulfates proportion. These results suggest that further public policy efforts that reduce fine particulate matter air pollution are likely to have continuing public health benefits.

## Supplemental Material

(197 KB) PDF

## References

[r1] Abbey DE, Nishino N, McDonnell WF, Burchette RJ, Knutsen SF, Lawrence Beeson W (1999). Long-term inhalable particles and other air pollutants related to mortality in nonsmokers.. Am J Respir Crit Care Med.

[r2] Akaike H (1973). Information theory and an extension of the maximum likelihood principle.

[r3] Anderson JO Thundiyil JG Stolbach A 2011 Clearing the air: a review of the effects of particulate matter air pollution on human health. J Med Toxicol; doi:10.1007/s13181-011-0203-1 [Online 23 December 2011] PMC3550231 22194192

[r4] Beelen R, Hoek G, van den Brandt PA, Goldbohm RA, Fischer P, Schouten LJ (2008a). Long-term exposure to traffic-related air pollution and lung cancer risk.. Epidemiology.

[r5] Beelen R, Hoek G, van den Brandt PA, Goldbohm RA, Fischer P, Schouten LJ (2008b). Long-term effects of traffic-related air pollution on mortality in a Dutch cohort (NLCS-AIR study).. Environ Health Perspect.

[r6] Beeson WL, Abbey DE, Knutsen SF (1998). Long-term concentrations of ambient air pollutants and incident lung cancer in California adults: results from the AHSMOG study. Adventist Health Study on Smog.. Environ Health Perspect.

[r7] Bennett WD, Zeman KL, Kim C, Mascarella J (1997). Enhanced deposition of fine particles in COPD patients spontaneously breathing at rest.. Inhal. Toxicol..

[r8] Cao J, Yang C, Li J, Chen R, Chen B, Gu D (2011). Association between long-term exposure to outdoor air pollution and mortality in China: a cohort study.. J Hazard Mater.

[r9] Dockery DW, Pope CA, Xu X, Spengler JD, Ware JH, Fay ME (1993). An association between air pollution and mortality in six U.S. cities.. N Engl J Med.

[r10] Dockery DW, Ware JH, Ferris BG, Glicksberg DS, Fay ME, Spiro A (1985). Distribution of forced expiratory volume in one second and forced vital capacity in healthy, white, adult never-smokers in six U.S. cities.. Am Rev Respir Dis.

[r11] Eftim SE, Samet JM, Janes H, McDermott A, Dominici F (2008). Fine particulate matter and mortality: a comparison of the six cities and American Cancer Society cohorts with a Medicare cohort.. Epidemiology.

[r12] Filleul L, Rondeau V, Vandentorren S, Le Moual N, Cantagrel A, Annesi-Maesano I (2005). Twenty five year mortality and air pollution: results from the French PAARC survey.. Occup Environ Med.

[r13] Gehring U, Heinrich J, Kramer U, Grote V, Hochadel M, Sugiri D (2006). Long-term exposure to ambient air pollution and cardiopulmonary mortality in women.. Epidemiology.

[r14] Grahame T, Schlesinger R. (2005). Evaluating the health risk from secondary sulfates in eastern North American regional ambient air particulate matter.. Inhal Toxicol.

[r15] Hernan MA (2010). The hazards of hazard ratios.. Epidemiology.

[r16] Katanoda K, Sobue T, Satoh H, Tajima K, Suzuki T, Nakatsuka H (2011). An association between long-term exposure to ambient air pollution and mortality from lung cancer and respiratory diseases in Japan.. J Epidemiol.

[r17] Kim SY, Sheppard L, Kim H (2009). Health effects of long-term air pollution: influence of exposure prediction methods.. Epidemiology.

[r18] Krewski D, Burnett RT, Goldberg M, Hoover K, Siemiatycki J, Abrahamowicz M (2005b). Reanalysis of the Harvard Six Cities study, part II: sensitivity analysis.. Inhal Toxicol.

[r19] Krewski D, Burnett R, Jerrett M, Pope CA, Rainham D, Calle E (2005a). Mortality and long-term exposure to ambient air pollution: ongoing analyses based on the American Cancer Society cohort.. J Toxicol Environ Health A.

[r20] Krewski D, Jerrett M, Burnett RT, Ma R, Hughes E, Shi Y (2009). Extended follow-up and spatial analysis of the American Cancer Society study linking particulate air pollution and mortality.. Res Rep Health Eff Inst.

[r21] Laden F, Neas LM, Dockery DW, Schwartz J (2000). Association of fine particulate matter from different sources with daily mortality in six U.S. cities.. Environ Health Perspect.

[r22] Laden F, Schwartz J, Speizer FE, Dockery DW (2006). Reduction in fine particulate air pollution and mortality: Extended follow-up of the Harvard Six Cities study.. Am J Respir Crit Care Med.

[r23] Laird N, Olivier D. (1981). Covariance analysis of censored survival data using log-linear analysis techniques.. J Am Statist Assoc.

[r24] Lepeule J, Caini F, Bottagisi S, Galineau J, Hulin A, Marquis N (2010). Maternal exposure to nitrogen dioxide during pregnancy and offspring birth weight: comparison of two exposure models.. Environ Health Perspect.

[r25] Lepeule J, Rondeau V, Filleul L, Dartigues JF (2006). Survival analysis to estimate association between short-term mortality and air pollution.. Environ Health Perspect.

[r26] McDonnell WF, Nishino-Ishikawa N, Petersen FF, Chen LH, Abbey DE (2000). Relationships of mortality with the fine and coarse fractions of long-term ambient PM_10_ concentrations in nonsmokers.. J Expo Anal Environ Epidemiol.

[r27] Miller KA, Siscovick DS, Sheppard L, Shepherd K, Sullivan JH, Anderson GL (2007). Long-term exposure to air pollution and incidence of cardiovascular events in women.. N Engl J Med.

[r28] Moolgavkar SH (2005). A review and critique of the EPA’s rationale for a fine particle standard.. Regul Toxicol Pharmacol.

[r29] Moolgavkar S. 2007 Pollution analysis flawed by statistical model [Letter] Nature 445 7123 21; doi:10.1038/445021c [Online 3 January 2007] 17203038

[r30] Nafstad P, Haheim LL, Wisloff T, Gram F, Oftedal B, Holme I (2004). Urban air pollution and mortality in a cohort of Norwegian men.. Environ Health Perspect.

[r31] Nehls GJ, Akland GG (1973). Procedures for handling aerometric data.. Journal of the Air Pollution Control Association.

[r32] Ostro B, Lipsett M, Reynolds P, Goldberg D, Hertz A, Garcia C (2010). Long-term exposure to constituents of fine particulate air pollution and mortality: results from the California Teachers Study.. Environ Health Perspect.

[r33] Pinkerton KE, Peake J, Plopper CG, Hyde DM, Tarkington BK (1995). Particles and the respiratory bronchiol: Patterns of deposition and clearance. Am J Resp Crit Care Med.

[r34] Pope CA, Burnett RT, Thun MJ, Calle EE, Krewski D, Ito K (2002). Lung cancer, cardiopulmonary mortality, and long-term exposure to fine particulate air pollution.. JAMA.

[r35] Pope CA, Burnett RT, Thurston GD, Thun MJ, Calle EE, Krewski D (2004). Cardiovascular mortality and long-term exposure to particulate air pollution: epidemiological evidence of general pathophysiological pathways of disease.. Circulation.

[r36] Pope CA, Rodermund DL, Gee MM (2007). Mortality effects of a copper smelter strike and reduced ambient sulfate particulate matter air pollution.. Environ Health Perspect.

[r37] Puett RC, Hart JE, Suh H, Mittleman M, Laden F (2011). Particulate Matter Exposures, Mortality, and Cardiovascular Disease in the Health Professionals Follow-up Study.. Environ Health Perspect.

[r38] Puett RC, Hart JE, Yanosky JD, Paciorek C, Schwartz J, Suh H (2009). Chronic fine and coarse particulate exposure, mortality, and coronary heart disease in the Nurses’ Health Study.. Environ Health Perspect.

[r39] Riva DR, Magalhaes CB, Lopes AA, Lancas T, Mauad T, Malm O (2011). Low dose of fine particulate matter (PM_2.5_) can induce acute oxidative stress, inflammation and pulmonary impairment in healthy mice.. Inhal Toxicol.

[r40] Schwartz J. (2000). The distributed lag between air pollution and daily deaths.. Epidemiology.

[r41] Schwartz J, Coull B, Laden F, Ryan L. (2008). The effect of dose and timing of dose on the association between airborne particles and survival.. Environ Health Perspect.

[r42] Szpiro AA, Paciorek CJ, Sheppard L (2011). Does more accurate exposure prediction necessarily improve health effect estimates?. Epidemiology.

[r43] Turner MC, Krewski D, Pope CA, Chen Y, Gapstur SM, Thun MJ (2011). Long-term ambient fine particulate matter air pollution and lung cancer in a large cohort of never-smokers.. Am J Respir Crit Care Med.

[r44] U.S. EPA (U.S. Environmental Protection Agency) (1997). Revisions to the National Ambient Air Quality Standards for Particulate Matter. Final rule.. Fed Reg.

[r45] U.S. EPA (U.S. Environmental Protection Agency) 2010 Regulatory Impact Analysis for the Proposed Federal Transport Rule. EPA-HQ-OAR-2009-0491. Research Triangle Park, NC:U.S. EPA.

[r46] U.S. EPA (U.S. Environmental Protection Agency) (2011). Air Trends in Particulate Matter.. http://www.epa.gov/airtrends/pm.html.

[r47] WHO (World Health Organization) (1977). Manual of the International Statistical Classification of Diseases, Injuries and Causes of Death. Ninth Revision.

[r48] WHO (World Health Organization) (1992). International Statistical Classification of Diseases and Related Health Problems. Tenth Revision.

[r49] Xu JQ, Kochanek KD, Murphy SL, Tejada-Vera B (2010). Deaths: Final Data for 2007. National Vital Statistics Reports.

[r50] Yorifuji T, Kashima S, Tsuda T, Takao S, Suzuki E, Doi H (2011). Long-term exposure to traffic-related air pollution and mortality in Shizuoka, Japan.. Occup Environ Med.

[r51] Zanobetti A Bind MA Schwartz J 2008 Particulate air pollution and survival in a COPD cohort. Environ Health 7 48; doi:10.1186/1476-069X-7-48 [Online 10 October 2008] 18847462 PMC2572050

[r52] Zanobetti A, Schwartz J. (2011). Ozone and survival in four cohorts with potentially predisposing diseases.. Am J Respir Crit Care Med.

