# Supplementary material for: Chronic Exposure to Fine Particles and Mortality: An Extended Follow-up of the Harvard Six Cities Study from 1974 to 2009
Source: Environ Health Perspect. 2012 Mar 28;120(7):965–70. doi: 10.1289/ehp.1104660 (PMC3404667; doi:10.1289/ehp.1104660)

**Supplemental Material**

Chronic Exposure to Fine Particles and Mortality: An Extended Follow-Up of the Harvard Six Cities Study from 1974 to 2009

Johanna Lepeule, Francine Laden, Douglas Dockery, Joel Schwartz.

**Table of contents:**

Supplemental Material, Table 1: Association between different $PM_{2.5}$ moving averages and mortality among participants who survived at least 5 years after enrolment (n=7805 participants, 180106 person-years 4209 deaths), Harvard Six Cities study, 1974-2009.

Supplemental Material, Figure 1: The estimated concentration-response fonction and 95% confidence interval between $PM_{2.5}$ and mortality in the Six Cities Study (1974-2009), using penalized splines.

**Supplemental Material, Table 1:** Association between different $PM_{2.5}$ moving averages and mortality among participants who survived at least 5 years after enrolment (n=7805 participants, 180106 person-years 4209 deaths), Harvard Six Cities study, 1974-2009.

| | | Cox regression[a] | | Poisson regression[b] | |
|---|---|---|---|---|---|
| Cause of death | $PM_{2.5}$ moving average (years) | Rate Ratio (95%CI) | AIC | Rate Ratio (95%CI) | AIC |
| All-cause | 1 | **1.15 (1.06-1.24)** | **31161.772** | **1.14 (1.05-1.23)** | **42347.57** |
| | 1-2 | 1.15 (1.06-1.23) | 31161.996 | 1.13 (1.05-1.22) | 42347.94 |
| | 1-3 | 1.14 (1.06-1.23) | 31161.907 | 1.13 (1.05-1.21) | 42347.92 |
| | 1-4 | 1.13 (1.05-1.21) | 31162.989 | 1.12 (1.04-1.20) | 42348.95 |
| | 1-5 | 1.13 (1.05-1.21) | 31162.675 | 1.12 (1.04-1.20) | 42348.74 |
| Cardio-vascular | 1 | **1.32 (1.17-1.48)** | **12369.478** | 1.31 (1.17-1.47) | 19739.23 |
| | 1-2 | 1.31 (1.17-1.47) | 12369.591 | 1.31 (1.17-1.46) | 19739.20 |
| | 1-3 | 1.30 (1.16-1.45) | 12369.546 | **1.30 (1.16-1.45)** | **19739.16** |
| | 1-4 | 1.29 (1.15-1.43) | 12370.363 | 1.28 (1.15-1.43) | 19740.06 |
| | 1-5 | 1.28 (1.15-1.42) | 12370.088 | 1.27 (1.15-1.41) | 19739.84 |
| Lung cancer | 1 | 1.28 (0.97-1.69) | 2229.420 | 1.38 (1.06-1.80) | 4751.03 |
| | 1-2 | 1.29 (0.98-1.70) | 2229.091 | **1.40 (1.07-1.82)** | **4750.41** |
| | 1-3 | **1.29 (0.99-1.69)** | **2229.013** | 1.39 (1.07-1.80) | 4750.51 |
| | 1-4 | 1.26 (0.97-1.64) | 2229.490 | 1.35 (1.05-1.74) | 4751.18 |
| | 1-5 | 1.24 (0.96-1.61) | 2229.696 | 1.33 (1.03-1.70) | 4751.61 |
| COPD | 1 | 1.18 (0.83-1.67) | 1568.151 | 1.12 (0.81-1.56) | 3600.23 |
| | 1-2 | 1.18 (0.84-1.68) | 1568.122 | **1.13 (0.81-1.58)** | **3600.20** |
| | 1-3 | 1.16 (0.83-1.64) | 1568.248 | 1.11 (0.80-1.54) | 3600.32 |
| | 1-4 | 1.17 (0.84-1.63) | 1568.166 | 1.11 (0.81-1.53) | 3600.28 |
| | 1-5 | **1.17 (0.85-1.62)** | **1568.105** | 1.11 (0.81-1.51) | 3600.29 |

AIC: Akaike Information Criterion

Results in bold are those with the lowest AIC value.

[a] Cox proportional hazard model stratified by sex, age, and time in the study and adjusted for body mass index, education, and smoking history

[b] Poisson survival analysis adjusted for sex, age, body mass index, education, smoking history, and time in the study (dummy variables for each year)

**Supplemental Material, Figure 1:** The estimated concentration-response fonction and 95% confidence interval between $PM_{2.5}$ and mortality in the Six Cities Study (1974-2009), using penalized splines.

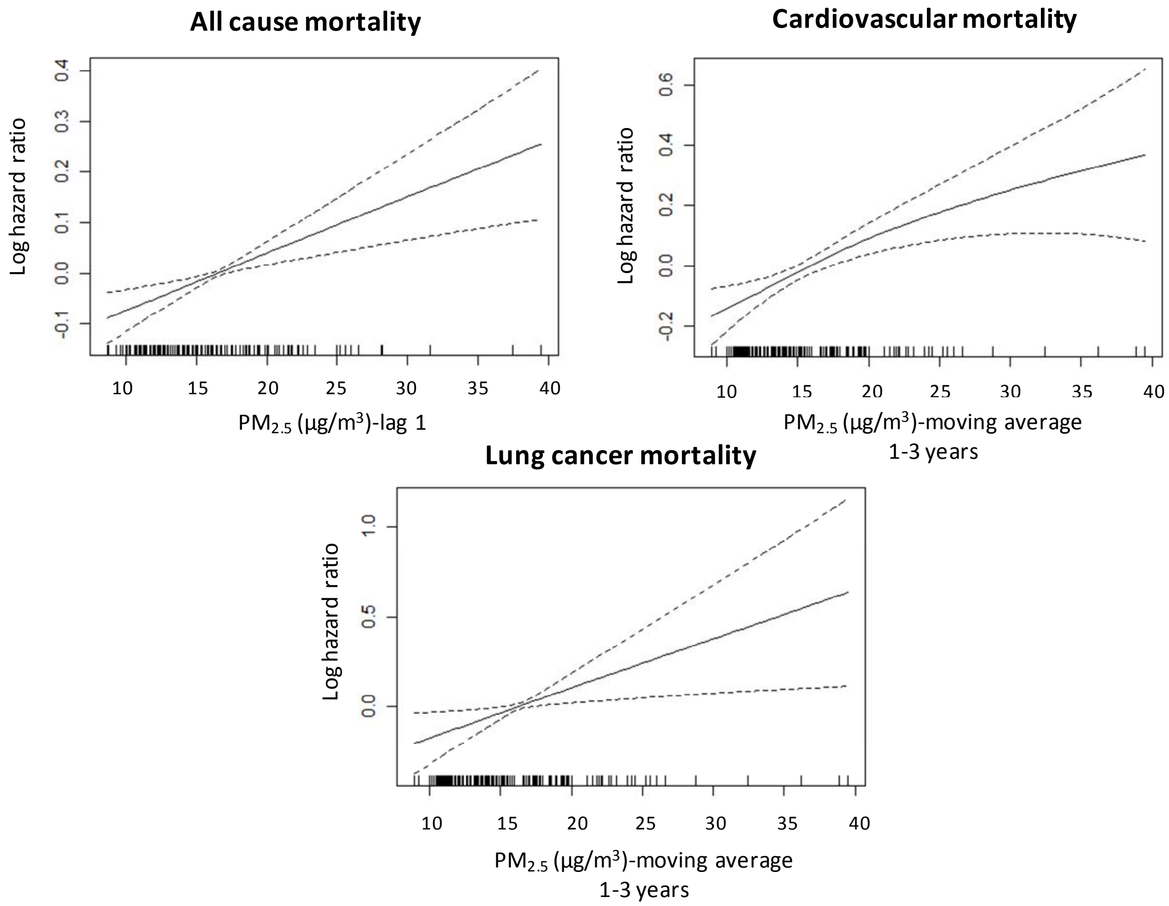

Supplement: (197 KB) PDF [file ehp.1104660.s001.pdf]
